# Alternative splicing signatures discriminate ATL cells from untransformed CD4+ counterparts deriving from HTLV-1 infected individuals

**DOI:** 10.1186/1742-4690-11-S1-O66

**Published:** 2014-01-07

**Authors:** Morgan Thenoz, Céline Vernin, Christiane Pinatel, Nicolas Nazaret, Joel Lachuer, Antoine Gessain, Didier Auboeuf, Eric Wattel, Franck Mortreux

**Affiliations:** 1 Oncovirologie et Biotherapies, UMR5239 CNRS/ENS Lyon/UCBL/HCL, Hopital Pierre Benite, Lyon, France; 2 Oncovirologie et Biothérapies, Centre Léon Bérard, Lyon, France; 3 ProfileXpert, Neurobiotec Service, Bron, France; 4 Unit of Epidemiology and Physiopathology of Oncogenic Viruses, Department of Virology, Institut Pasteur, Paris, France; 5 Institut National de Santé et de Recherche Médicale U590, Centre Léon Bérard, Lyon, France

## 

The clonal expansion and malignant transformation of HTLV-1 infected CD4+ T-cells have been linked to the reprogramming effects of HTLV-1 on host transcriptional profile. Coupled to transcription, alternative splicing (AS) is a post-transcriptional process that plays critical role in the complexity of transcriptome and splicing abnormalities frequently occur in cancer. To examine whether AS modifications associate with HTLV-1-associated leukemogenesis, we compared the exon expression profiles of ATL cells with that of CD4+ T-cell clones obtained by limited-dilution cloning of PBMC deriving from HTLV-1 carriers. 3 ATL cells and 12 untransformed infected clones clustering in infected, uninfected, PHA-stimulated or unstimulated CD4+ T cells were compared for exon RNA content using Exon Chip Human microarray. Hierarchical clustering analysis identified 12516 alternative spliced events (3642 genes) that clearly separated ATL samples from the 4 untransformed phenotypes mentioned above. In contrast, the exon content of 1539 genes differed between untransformed infected and uninfected T-CD4+ cells. Overall, less than 5% alternatively spliced genes were found differentially expressed at the transcriptional level. Microarray data were confirmed for 18 AS events using exon specific RT-PCR analysis. Pathway analysis of alternatively spliced genes (3642) in ATL cells revealed new AS-based pathways for p53 signaling, cell cycle and DNA replication while those of untransformed infected CD4+ T-cells were enriched in pathways for cellular movement and DNA repair. These findings unveil a new layer of complexity in the interplay between HTLV-1 and host cell gene expression machinery in which AS might play a central role in tumor initiation and promotion.

